# Variation in volatile organic compounds in Atlantic salmon mucus is associated with resistance to salmon lice infection

**DOI:** 10.1038/s41598-022-08872-z

**Published:** 2022-03-22

**Authors:** G. F. Difford, J.-E. Haugen, M. L. Aslam, L. H. Johansen, M. W. Breiland, B. Hillestad, M. Baranski, S. Boison, H. Moghadam, C. Jacq

**Affiliations:** 1 Breeding and Genetics Nofima, Norwegian Institute of Food, Fisheries and Aquaculture Research, Osloveien 1, 1430 Ås, Norway; 2 Food and Health Nofima, Norwegian Institute for Food, Fisheries and Aquaculture Research, Osloveien 1, 1430 Ås, Norway; 3 Fish Health Nofima, Norwegian Institute for Food, Fisheries and Aquaculture Research, Muninbakken 9, 9019 Tromsø, Norway; 4 Benchmark Genetics Norway AS, Sandviksboder 3A, Bergen, Norway; 5 Mowi Genetics AS, Sandviksboder 77AB, Bergen, Norway; 6 Viking Aqua AS, Sandevegen 631, 5997 Ånneland, Norway; 7 Blue Analytics AS, Kong Christian Frederiks plass 3, 5006 Bergen, Norway

**Keywords:** Genetics, Agricultural genetics, Animal breeding, Quantitative trait

## Abstract

Salmon lice are ectoparasites that threaten wild and farmed salmonids. Artificial selection of salmon for resistance to the infectious copepodid lice stage currently relies on in vivo challenge trials on thousands of salmon a year. We challenged 5750 salmon with salmon lice (*Lepeophtheirus salmonis*) from two distinct farmed strains of salmon in two separate trials. We found that volatile organic compounds (VOC), 1-penten-3-ol, 1-octen-3-ol and 6-methyl-5-hepten-2-one in the mucus of the salmon host after salmon lice infection, were significantly associated with lice infection numbers across a range of water temperatures (5 °C, 10 °C, 17 °C). Some VOCs (benzene, 1-octen-3-ol and 3,5,5-trimethyl-2-hexene) were significantly different between lines divergently selected for salmon lice resistance. In a combined population assessment, selected VOCs varied between families in the range of 47- 59% indicating a genetic component and were positively correlated to the salmon hosts estimated breeding values 0.59–0.74. Mucosal VOC phenotypes could supplement current breeding practices and have the potential to be a more direct and ethical proxy for salmon lice resistance provided they can be measured prior to lice infestation.

## Introduction

According to fossil records, copepods have been parasitizing fish for at least the last 100 Ma or longer^1^. Salmon lice (*Lepeophtheirus salmonis*) are no exception, with the Atlantic subspecies of salmon lice having coevolved to parasitize Atlantic salmon (*Salmo salar*) hosts over the last (c 2.5–11 Ma)^2^. Host-parasite co-evolution is typically a rapid and reciprocal process where each species counter adapts the other’s defences, essentially amounting to a zero-sum game (i.e. Red Queen hypothesis)^3^. Since the intensification of Atlantic salmon farming in the second half of the twentieth century, salmon lice have become an intractable problem, with devastating effects on both wild and captive Atlantic salmon^4^. Chemical treatments have been followed by rapid and successive adaptation and resistance of salmon lice^5–8^, suggesting that repeated reintroduction of naïve salmon at high densities in sea cages may disproportionately favour parasitic adaptation. Strategies aimed at preventing the parasite from attaching to salmon have shown the most promise in terms of breaking the host-parasite coevolutionary arms race^6,8^. A fundamental question is whether it is possible to re-establish a steady-state host-parasite equilibrium that ensures sustainable and ethical Atlantic salmon production.

Previous research focused on comparing and elucidating the large differences in post-attachment resistance to salmon lice between Atlantic salmon and Pacific salmon species belonging to the genus Oncorhynchus^9–11^. Interestingly, in standardised in vivo challenge trials, the initial numbers of attached salmon lice are similar between Atlantic salmon, and Pacific salmon species^12^. However, the more resistant coho (*O*. *kisutch*) and pink salmon (*O*. *gorbuscha*) rapidly reject lice a few days post attachment^13^. In coho salmon, this phenomena has been linked to a rapid and pronounced non-specific epithelial hyperplasia and cellular infiltration responses which result in rejection of the parasite a few days after attachment^14^.Conversely, pink salmon respond with a localised inflammatory response and strong iron sequestration^11^. These findings have sparked interest in the introgression of resistance genes from Pacific salmon species into Atlantic salmon, but this technology is in its formative stages and key genomic information on these pathways is still needed^15,16^.

Artificial selection for host resistance to attachment by the free-living larval phase (copepodids) presents a strong approach as it reduces encounter rates and inhibits completion of the parasitic lifecycle^6,15^. In principle, salmon lice could counter-adapt to artificial selection methods for host resistance. However, pathogen counter-adaptation to polygenic host resistance in plant and livestock systems (including salmonids) has been far slower than counter-adaptation to chemical treatments^8^. Suggesting that artificial selection for host resistance could be an effective method to establish a host-parasite equilibrium at a steady state. Regardless, identifying genetically elite Atlantic salmon, relies on standardised in vivo parasite challenge trials on thousands of informant fish (siblings to breeding candidates) each generation. The informant fish are manually counted for copepodite attachment by trained observers post-mortem, thus they cannot be used for selective breeding directly. Compared to selective breeding where breeding candidates have their own phenotype, this indirect method of selection on deceased informant relatives is less efficient, in addition to the serious logistical and ethical limitations to challenge testing. All the while, the underlying molecular basis for host resistance to parasite attachment remains undescribed.

The key to salmon lice resistance may lie in circumventing how the free-swimming copepodid identifies a suitable host in a vast ocean environment. Kairomones are semiochemical compounds emitted by an organism, which provide a direct benefit to another organism, often to the detriment of the emitting organism. Kairomones that govern host detection in biting insects such as mosquitoes are well-described and are predominantly volatile organic compounds (VOCs)^17^. Yet, the importance of understanding the roles of VOC kairomones in aquatic species has only recently been recognized^18^. Behavioural assays and electrophysiological experiments indicated that adult male salmon lice were attracted to three VOCs, namely phenol, 3,5,5-trimethylcyclohex-2-en-1-one (isophorone) and 1-octen-3-ol found in salmon conditioned water^19^. A separate study found that salmon lice copepodids were attracted to two VOCs: 3,5,5-trimethylcyclohex-2-en-1-one and 6-methyl-5-hepten-2-one (sulcatone)^20^. Further studies characterized the activation of copepodid antennules to salmon-conditioned water containing VOCs and the induced positive rheotactic swimming behaviours^21,22^. Komisarczuk et al.,^23^ identified ionotropic receptors highly expressed in the antennules of salmon lice copepodids and when the specific ionotropic receptors were knocked down, the copepodids demonstrated impaired host-seeking and settlement abilities. Together, these findings suggest Atlantic salmon VOC kairomones aid copepodids in seeking a host. However, there is, as yet, no established link between lice infection in genetic in vivo challenges and mucosal VOCs.

To this end, we screened 5750 Atlantic salmon from two distinct farmed strains for resistance to copepodid infection in two separate challenge trials. The first strain represented a broad genetic base with no documented artificial selection for resistance against salmon lice, with the challenge test performed across a range of temperatures representing the thermal spectrum for Atlantic salmon farming (5 °C, 10 °C, 17 °C) (Temp). The second strain represented divergent genetic lines after three generations of selection for salmon lice resistance, representing high resistance (GenR) and low lice resistant (GenS) lines (Gen). In Temp we sampled the skin mucus from the 1% of fish with the highest (n = 24) and the lowest (n = 24) copepodid infection in each tank. In Gen we randomly sampled mucus from 10% (n = 24) fish across families within divergent lines.

Using these data, we address the following questions: (1) Are mucosal VOCs associated with copepodid infectivity across the thermal tolerance of Atlantic salmon? (2) Do divergent genetic lines for lice infection have differential expression of VOCs? (3) Is there evidence of genetic variation for VOC expression and salmon lice resistance? Overall, our goal was to understand the molecular mechanisms of salmon lice resistance and identify compounds for future directed phenotypes.

## Results

We found two VOCs previously implicated in host seeking behaviour of salmon lice (1-octen-3-ol^24^ and 6-methyl-5-hepten-2-one^20^) in our two populations. Interestingly, the mucosal VOCs expressed were not identical across the Temp and Gen trials, with only two VOCs co-occurring across both trials (1-octen-3-ol and 6-methyl-5-hepten-2-one), two VOCs only occurring in Gen (3,5,5-trimethylcyclohex-2-en-1-one and benzene), and one exclusively occurring in Temp (1-penten-3-ol). In addition, phenol and 3,5,5-trimethylcyclohex-2-en-1-one^20,24^ which were previously implicated as kairomones^24^, were absent from all our samples, but benzene (which differs to phenol by a functional group) and 3,5,5-trimethyl-2-hexene were present in the Gen trial. In addition, 1-penten-3-ol was co-abundant with 1-octen-3-ol in the Temp trial and retained for further analysis.

We observed co-occurrence of two of the five VOCs differentially expressed across both populations’ mucosal VOC bouquets. Despite both strains being cultured under the same conditions and on the same diet, we observed differential occurrence between the strains for compounds. We found the previously reported kairomones 6-methyl-5-hepten-2-one^17,20^ and 1-octen-3-ol^19,26^ in both strains. We did not observe the previously reported phenol or 3,5,5-trimethylcyclohex-2-en-1-one^19,20^ in either of the strains. The novel compound 1-penten-3-ol was, however, only found in the Temp trial whilst benzene and 3,5,5-trimethyl-2-hexene were only found in the Gen trial.

**Figure 1:**
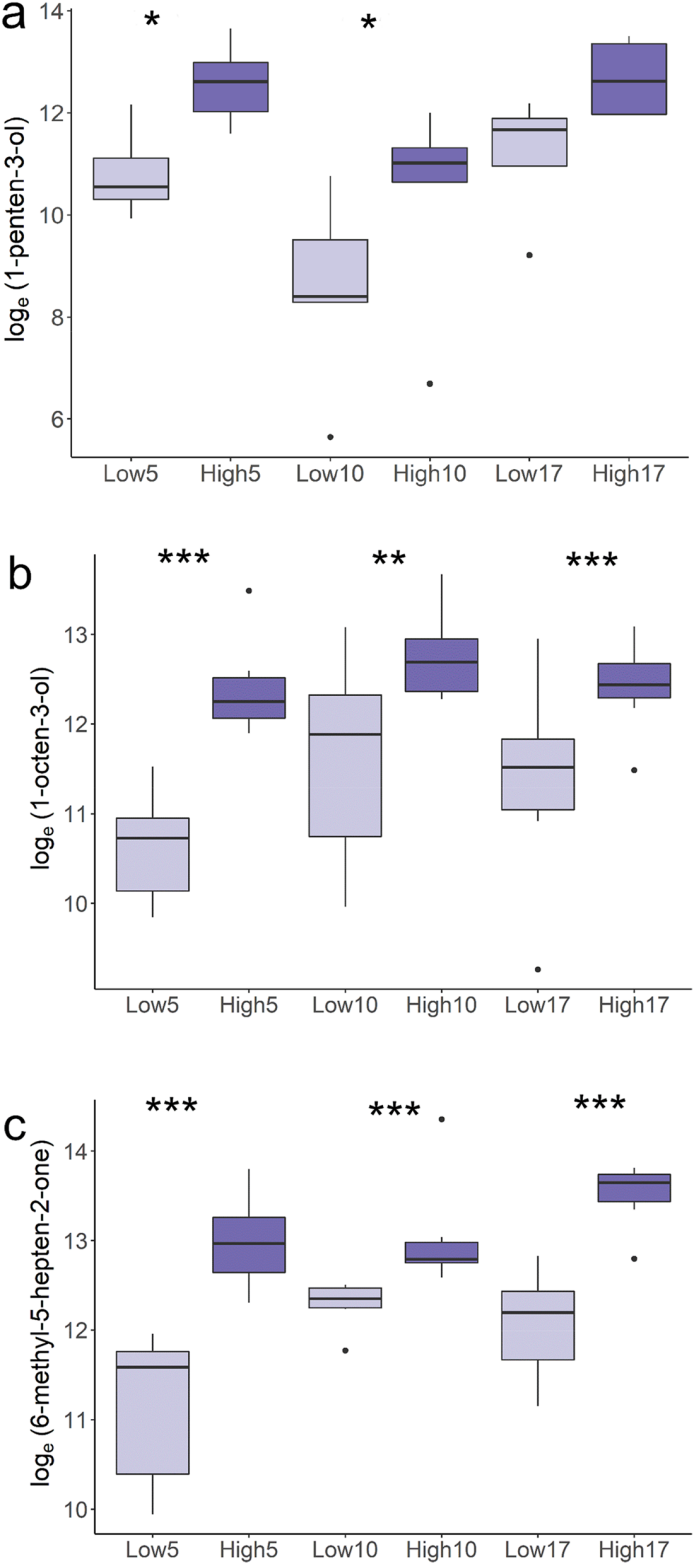
Boxplot and whisker plot showing differential expression (Loge to GC peak area) of 1-penten-3-ol (**a**), 1-octen-3-ol (**b**) and 6-methyl-5-hepten-2-one (**c**) across salmon with high and low lice count (High, Low) across culture temperatures (5, 10 and 17 °C), n = 4 per group, significance level after Benjamini–Hochberg correction (*p < 0.05; **p < 0.01; ***p < 0.001). Median is given as solid line, box is the interquartile range and whiskers are 1.5 times the interquartile range.

Across all three water temperature challenges, VOCs were significantly expressed in fish with high lice infection (ANOVA 1df p < 0.001) (Fig. 1a–c and Table 1), strengthening the assertion that VOC expression is modulated by the host and not a response to water temperature-induced metabolic rate. Although the effect of water temperature was significant for all three VOCs in Temp (ANOVA, 4 df, p < 0.001), there was no significant interaction term between VOC expression levels in high and low lice infection fish across water temperature. 6-methyl-5-hepten-2-one did display a trend of increasing expression with increasing water temperature (Fig. 1c).

**Table 1:** Effects of water temperature and high or low lice group on 1-penten-3-ol, 1-octen-3-ol and 6-methyl-5-hepten-2-one mucosal expression levels during salmon lice infection.

Water temperature	5 °C		10 °C		17 °C		Significant Water temperature	Significance lice group	R^2^
Lice group**	Low Lice	High Lice	Low Lice	High Lice	Low Lice	High Lice			
Loge(1-penten-3-ol)***	10.8^a^ ± 0.5	12.6^be^ ± 0.6	8.56^c^ ± 0.5	10.3^abf^ ± 0.6	11.2^ab^ ± 0.7	12.7^be^ ± 0.6	P < 0.001	P < 0.001	0.52
Loge (1-octen-3-ol)***	10.6^a^ ± 0.3	12.4^c^ ± 0.3	11.7^b^ ± 0.3	12.8^c^ ± 0.3	11.3^ab^ ± 0.3	12.4^c^ ± 0.3	P < 0.05	P < 0.001	0.99
Loge(6-methyl-5-hepten-2-one)***	11.1^a^ ± 0.2	13.0^c^ ± 0.2	12.2^b^ ± 0.2	13.1^c^ ± 0.2	12.1^b^ ± 0.2	13.5^c^ ± 0.2	P < 0.001	P < 0.001	0.63

*Different superscripts within row differ significantly at P < 0.05,** High and low lice groups correspond to the two highest and two lowest ranked fish within each tank replicated in each temperature treatment, ** VOC measured as integrated GC peak area, standard errors of estimates given after estimates ±.

**Figure 2:**
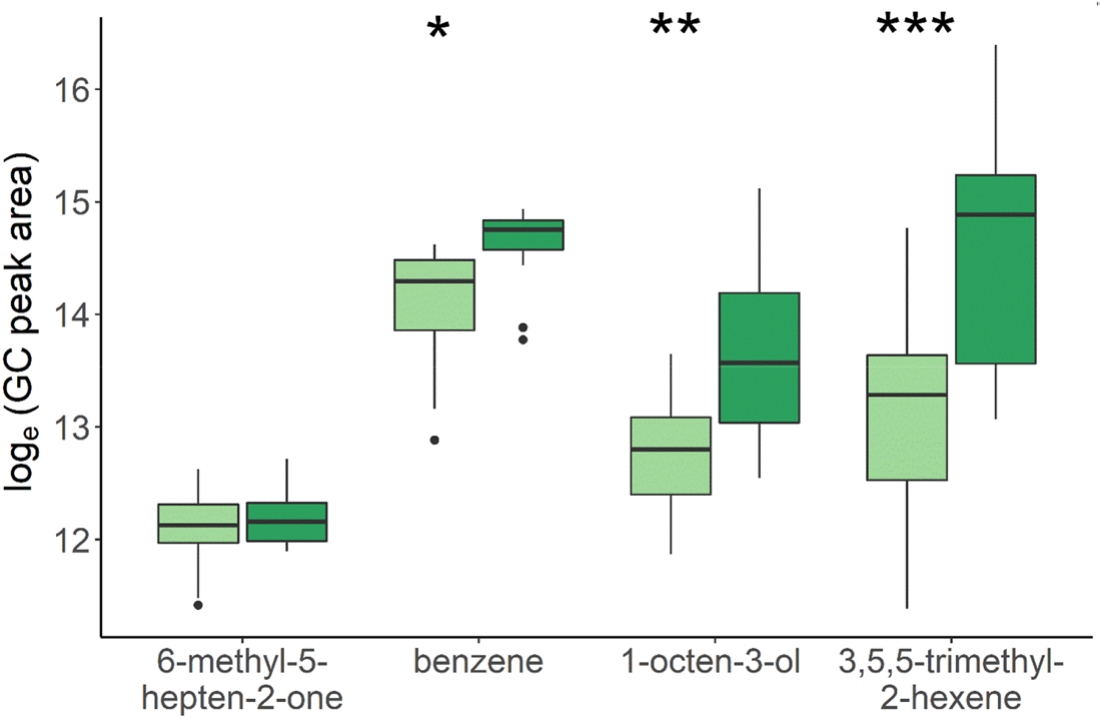
Boxplot and whisker plot showing differential expression (Loge to GC peak area) of 6-methyl-5-hepten-2-one, benzene, 1-octen-3-ol and 3,5,5-trimethyl-2-hexene (c) across genetically resistant GenR (light green n = 12) and susceptible GenS (dark green n = 12) lines, significance level after Benjamini–Hochberg correction (*p < 0.05; **p < 0.01; ***p < 0.001). Median is given as solid line, box is the interquartile range and whiskers are 1.5 times the interquartile range.

The genetically susceptible (GenS) and resistant (GenR) lines of Atlantic salmon had significantly different copepodite counts after in vivo challenges (ANOVA, df = 1, P < 0.001) (Table 2). The resistant strain having on average 10 less copepodites per individual amounting to a difference in means of 40% on the observed count scale, demonstrating a realised response to selection for lice resistance. Significant differential expression was also observed for 1-octen-3-ol, 3,5,5-trimethyl-2-hexene and benzene, between the divergent genetic lines for lice resistance (Fig. 2). Indicating selection for resistance against salmon lice has resulted in a correlated response in mucosal VOC composition. No significant difference was observed for 6-methyl-5-hepten-2-one between GenR and GenS lines. A single control sample from the water inlet detected lower levels of benzene and none of the other VOCs. Samples from mixed GenS and GenR fish prior to salmon lice infestation (n = 10) detected 1-octen-3-ol, 3,5,5-trimethyl-2-hexene and benzene at marginally lower levels than post lice infestation, whilst 6-methyl-5-hepten-2-one was detected at marginally higher levels than post infection (Table 1).

**Table 2:** The effect of Genetic lines susceptible (GenS) and resistance (GenR) to salmon lice infection on lice counts and mucosal VOC expression.

Trait	Control – water inlet	Control – mucus prior to infection	N	GENS	GENR	Significance level	R^2^
Log10(Lice)	N/A	N/A	171	1.48 ± 0.02	1.32 ± 0.02	P < 0.001	0.97
Loge(1-octen-3-ol)	0	10.4 ± 0.2	24	13.7 ± 0.2	12.8 ± 0.2	P < 0.005	0.99
Loge(6-methyl-5-hepten-2-one)	0	12.8 ± 0.1	24	12.2 ± 0.1	12.1 ± 0.1	P < 0.05	0.99
Loge(3,5,5-trimethyl-2-hexene )	0	12.0 ± 0.1	24	14.6 ± 0.3	13.1 ± 0.3	P < = 0.001	0.99
Loge(benzene)	9.2	11.3 ± 0.1	24	14.6 ± 0.1	14.1 ± 0.1	P < 0.05	0.99

Trait – Lice is count, VOC measured as integrated GC peak area, Control – water inlet = VOC in a single water sample from the inlet, Control – mucus prior to infection = VOC in 10 random samples before the fish were challenged with lice, N = number of fish sampled, GenS = the genetically susceptible line, GenR = genetically resistant line. R^2^ = coefficient of determination. Standard errors given after estimates and  ± .

Quantitative genetic analysis revealed that lice infection is significantly heritable across populations (h^2^ = 0.23) (Table 3) as reported previsouly^25,27–29^ and identified the host genetic potential to resist lice infection with high reliability of estimated breeding values. There was also significant heritability in the broad sense which revealed that family variation explained between 47 and 59% of the variation in 1-octen-3-ol, 3,5,5-trimethyl-2-hexene and benzene across populations (Table 3). The phenotypic expression of 1-octen-3-ol and 3,5,5-trimethyl-2-hexene were also correlated to the host genetic potential to resist copepodite infection (estimated breeding values) (r = 0.59 – 0.74).

**Table 3:** Genetic parameter estimates of mucosal VOCs and their phenotypic correlation to salmon lice resistance estimated breeding values.

	N	Trial	Additive genetic variation	Phenotypic variation	Narrow sense heritability	Correlation to host genetic potential for resistance
Log10(Lice)	5749	Combined	0.087^a^	0.381	0.23* ± 0.05	1
	N	Trial	Between family genetic variation	Phenotypic variation	Broad sense heritability	Correlation to host genetic potential for resistance
Loge(1-penten-3-ol)	48	Temp	N/A	2.40	N/A	0.65 ± 0.11
Loge(1-octen-3-ol)	72	Combined	0.573	0.997	0.58 ± 0.17	0.59 ± 0.11
Loge(3,5,5-trimethyl-2-hexene )	24	Gen	0.473	1.001	0.47 ± 0.23	0.74 ± 0.14
Loge(6-methyl-5-hepten-2-one)	72	Combined	0.462	1.214	0.37 ± 0.20	0.50 ± 0.10
Loge(benzene)	24	Gen	0.132	0.225	0.59 ± 0.20	0.37 ± 0.20

*N = number of fish in analysis, N/A = not applicable, values after ± are standard errors.

## Discussion

**Figure 3:**
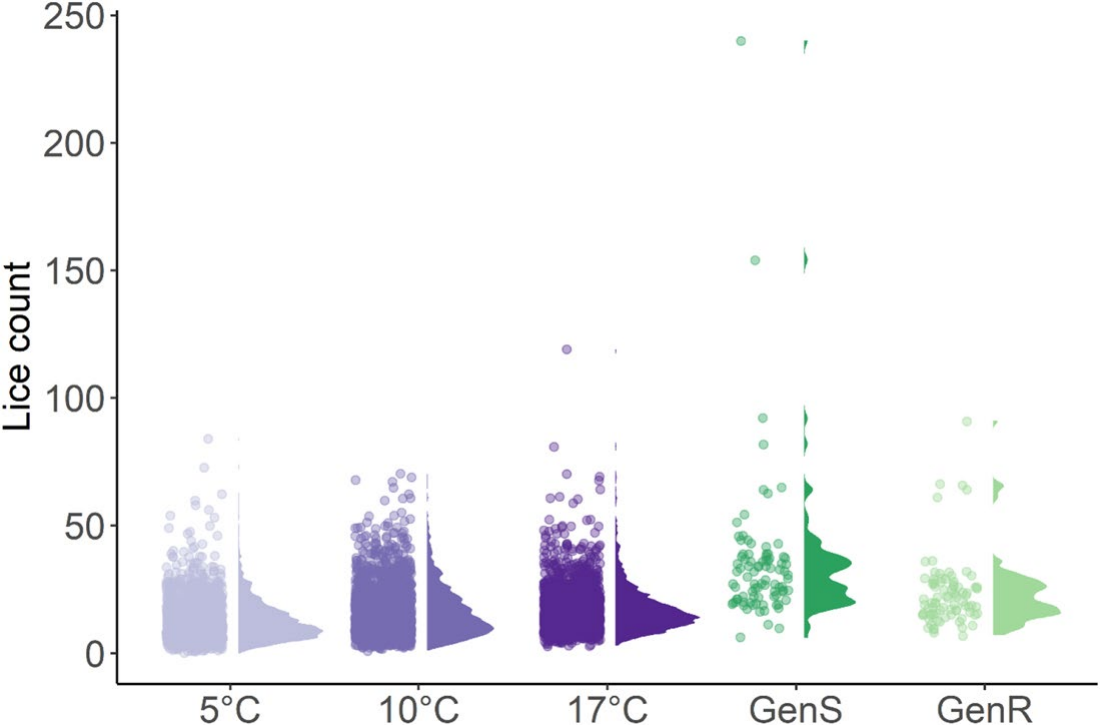
Salmon lice counts per fish shown as paired point scatter and distribution plots across the three water temperature groups (5 °C, 10 °C, 17 °C) in the Temp trial (shades of purple following temperature gradient) and the susceptible strain (GenS in dark green) and resistant strain (GenR in light green) in the Gen trial.

**Figure 4:**
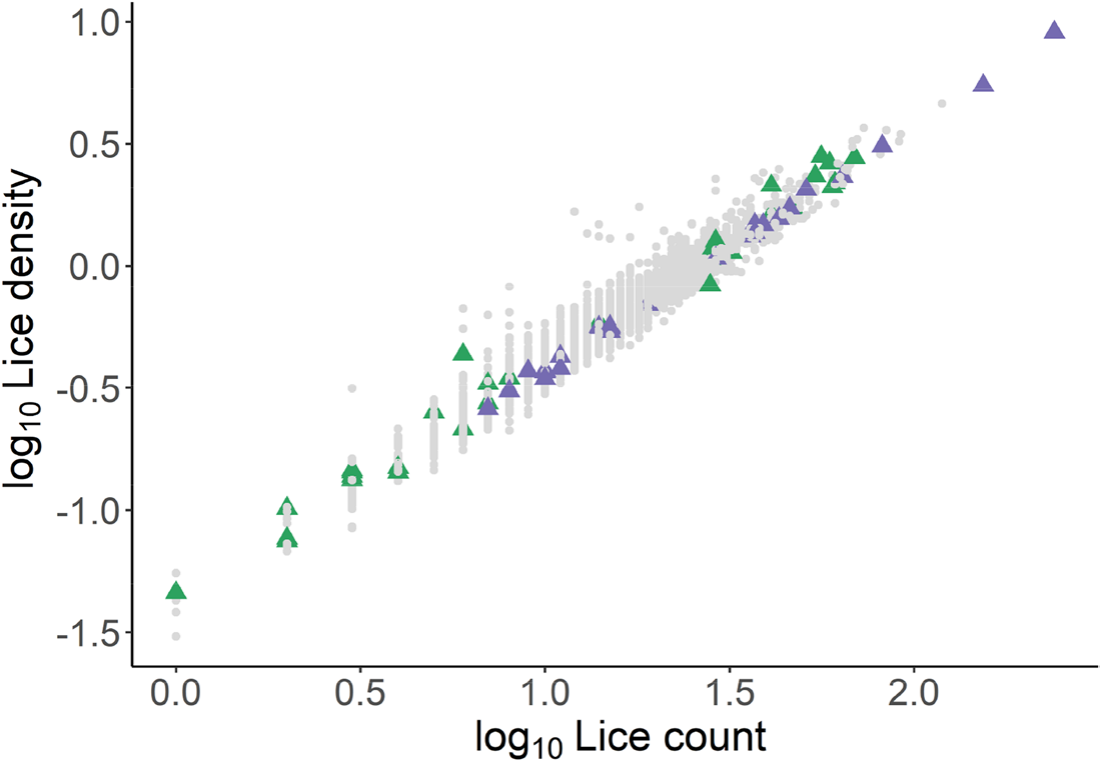
Linear relationship between lice count per host and lice density (count per unit surface area estimated as lice count/body weight^2/3^)^25^ across both Temp and Gen trials. Samples selected for VOC analysis are given by triangle, green denoting Gen and purple denoting Temp.

Across all copepodite challenges, large differences in infectivity between individuals were observed (Fig. 3), independent of host size, indicating that larger fish did not attract more lice within the size ranges in our trials (Fig. 4). The average of the log10 transformed lice counts were not significantly different between the challenge trials Temp and Gen (Supplementary Table 1), demonstrating good reproducibility over trials and bath challenges.

The fish in both trials were cultured at the same facility, on the same diet, in the same water resources and challenged with the same salmon lice at the same body size and life stage. Thus, the differential presence of VOCs across the two strains used in the Gen and Temp trials raises further questions about strain specific kairomones or the possibilities for early dietary influences or maternal influences through egg composition or the factors affecting the substrates from which VOCs are derived. For instance, 1-penten-3-ol and 1-octen-3-ol being secondary lipid oxidation products of long chain fatty acids, are abundant in the muscle of salmonids and other fish and used as freshness markers^30^. Studies have found Atlantic salmon from east and west Europe and rainbow trout (*O. mykiss*) express significantly different amounts of both compounds in their muscle, with some VOCs absent from either salmon or rainbow trout populations^31^. In addition, expression of 1-octen-3-ol changes over the Atlantic salmon life cycle, with significantly greater expression in Atlantic salmon during early sea phase compared to the freshwater phase^32^, which corresponds to the timing of exposure to salmon lice in wild and farmed salmon. Whilst 1-penten-3-ol expression increased in the later sea phases^32^. If mucosal expression of 1-octen-3-ol and 1-penten-3-ol follows expression patterns in salmon muscle, then mucosal expression may also be affected by species, strains, life stages and diets. What is currently not known is to what extent salmon lice rely on VOCs collectively for identification of a suitable host and to what extent specific VOCs are redundant in lice attraction to the host.

We investigated the effects of water temperature and the association between high and low lice infection with VOCs expression levels. A previous study found salmon lice to be most successful at infesting Atlantic salmon at 10 °C (percentage successful copepodid settlement 53.2%), followed by 20 °C (41.6%) and 5 °C (2.1%)^33^, suggesting that the host parasite interaction might be modulated by an optimal water temperature around 10 °C. We found that across all three water temperatures (5 °C, 10 °C and 17 °C) the association between greater expression of 1-penten-3-ol, 1-octen-3-ol and 6-methyl-5-hepten-2-one and high lice infection remained significant. However, kairomones 1-octen-3-ol and 6-methyl-5-hepten-2-one had significantly lower expression in the low lice infected groups at lower water temperatures, offering a possible explanation for poor infestation success at 5 °C reported previously^33^. Interestingly, 6-methyl-5-hepten-2-one showed an increasing trend for expression levels with increasing water temperatures suggesting this VOC may become increasingly important in host-parasite interactions under scenarios of increasing sea water temperatures. All three compounds are secondary oxidation products with 1-penten-3-ol, 1-octen-3-ol resulting from oxidation of long chain fatty acids like linoleic acid, arachidonic acid and eicosapentaenoic acid which are themselves heritable in Atlantic salmon^34–36^. Whilst, 6-methyl-5-hepten-2-one results from the oxidation of terpenoid compounds like astaxanthin and squalene responsible for pigmentation in Atlantic salmon and similarly also heritable compouds^37–39^. Increasing water temperatures result in linear increases in mitochondrial respiration and production of reactive oxidative species in Atlantic salmon^40^, which have been linked to altered fatty acid composition^41^ and pigmentation depletion^42^. Thus, increasing water temperatures and the role of oxidative stress metabolism may contribute to the production of VOCs through secondary lipid and terpenoid oxidation.

Importantly, salmon lice resistance is a highly variable quantitative trait with a highly polygenic genetic component (h^2^ = 0.23)^43,44^, thus genetically divergent strains selected for or against salmon lice resistance are not completely or categorically resistant or susceptible. Rather they display a spectrum of lice counts with resistant strains having lower counts than more susceptible strains. In practice, salmon lice resistance is one of many traits selected for in each generation, depending on the relative importance it is given in the breeding goal, future generations will continue to show quantitative improvements to salmon lice resistance and thus decreasing lice counts. In the Gen trial we found that the GenR strain had significantly lower lice infestation than the GenS strain, corresponding to 10 less lice per fish on average, demonstrating to the best of our knowledge, the first evidence of realised response to selection against lice infestation. Differences between divergently selected lines provide insight into correlated or indirect responses to selection. The expression of mucosal VOCs benzene, 1-octen-3-ol and 3,5,5-trimethyl-2-hexene were significantly different between the GenR and GenS, suggesting that response to selection for resistance to salmon lice may have resulted in an indirect response for decreased expression of these VOCs. Surprisingly, 6-methyl-5-hepten-2-one expression did not differ between the GenR and GenS strains, despite being significantly associated with lice infestation in the Temp trial. Observed differences in traits between divergent selection lines suggest that benzene, 1-octen-3-ol and 3,5,5-trimethyl-2-hexene may be influenced by host genetics and conversely, 6-methyl-5-hepten-2-one may be influenced to a lesser extent, or not at all by host genetics. This is further supported by the broad sense heritability estimates, which showed that the differences between families of Atlantic salmon explained large proportions (47- 59%) of the variation in benzene, 1-octen-3-ol and 3,5,5-trimethyl-2-hexene. Whilst the broad sense heritability of 6-methyl-5-hepten-2-one was a moderate 37% and not significantly different from 0, indicating limited genetic influence. The correlation between the estimated breeding values for lice infection and VOC expression further establishes covariation between host genetics, VOC expression and sea lice infection. With strong positive correlations ranging from 0.59 – 0.74 between 1-penten-3-ol, 1-octen-3-ol and 3,5,5-trimethyl-2-hexene and salmon lice resistance, whilst 6-methyl-5-hepten-2-one and benzene had modest positive correlations ranging from 0.37 – 0.50.

A limitation of the present study is that mucus samples associated with lice counts were taken after salmon lice infestation and thus an important assumption is that mucosal VOC expression is not influenced by salmon lice infestation. This assumption may not entirely hold, as studies have found that salmon lice have the ability to alter the protein mucosal composition of the host in attempts to immunomodulate the host^13,45^ and it is not known if they can alter the VOC expression. In the Gen trial we found 1-octen-3-ol, 6-methyl-5-hepten-2-one, benzene and 3,5,5-trimethyl-2-hexene expressed at similar albeit generally lower levels prior to sea lice infestation and all were absent from the inlet water, with the exception of benzene which was found at lower levels. This gives some evidence that mucosal VOCs analysed herein were expressed prior to sea lice infestation and the associations between lice count and VOC expression are not likely to be entirely due to salmon lice altering host mucus composition. However, if salmon lice do alter the mucosal VOC bouquets of infected salmon, then associations maybe driven because of the magnitude of lice infection and not cause infection. For some of the VOCs (1-octen-3-ol, and 6-methyl-5-hepten-2-one) their kairomone activity and role as lice attractants has been firmly established in behavioural and electrophysiological experiments previsouly^20,24^. However, the associations between lice infection with 1-penten-3-ol, 3,5,5-trimethyl-2-hexene and benzene need further validation before their role as attractants can be established. In addition, a number of the VOCs identified are secondary lipid and terpenoid oxidation products which can be triggered by environmental stressors such as water temperature. Lice attachment can also be considered an external stressor and potentially induce an oxidative stress response in salmon skin, triggering the generation of reactive oxygen species and consequently increased VOCs. Thereby establishing a positive feedback loop which promotes further lipid and terpenoid oxidation and consequently the production of more volatile secondary lipid oxidation products with kairomone activity further attracting more copepodites. Indeed, a recent review has identified trends of increased oxidative stress responses in the skin of salmonids infected with different lice species including salmon lice^46^. Future investigations into genetic variation in mucosal VOC prior to salmon lice infection are needed to further elucidate these mechanisms.

Lastly, we sampled modest numbers (ranging from 24 to 72) of carefully selected fish for VOC analysis and employed a number of genetic approaches to established evidence for genetic differences in VOC expression and lice infection, including comparing divergent genetic lines, quantifying between family variation and calculating correlations to the estimated breeding values for lice infection in a large cohort of Atlantic salmon (5750). However, far larger numbers of fish need to be recorded for mucosal VOC composition in order to accurately quantify the additive genetic component and narrow sense heritability of VOC expression. One of the most promising compounds found across trials and with evidence of host genetic variation (1-octen-3-ol) happens to also be produced by humans and is of interest as a cancer biomarker and mosquito attractant, consequently developments in rapid and sensitive octenol biosensors technologies^47^, may benefit VOC measurement in Atlantic salmon in the future. If the measurement of inherent mucosal VOC profiles on individual breeding candidates can be done on a large scale, non-invasively and prior to salmon lice infestation and shows significant additive genetic variation, it has enormous potential to enable more efficient direct selection for lice resistance and as an ethical alternative to copepodid lice challenges.

## Methods

### Ethical statement

All experiments were carried out in strict accordance with relevant guidelines and regulations. The study was carried out with approval granted from the Ethic committee of *Norwegian Food Safety Authority* (approval numbers 13569 Temp and 13571 Gen) following ARRIVE guidelines.

### Salmon lice in vivo infection challenges

Two populations of Atlantic salmon smolts from Benchmark Genetics (Gen) and MOWI strains (Temp) were challenged with *L.salmonis* copepodids at Aquaculture Research Station in Tromsø (Tromsø, Norway). The salmon lice used were the Atlantic subspecies of *L. salmonis* originating from the mixing of the LsOslo and LsGulen strain from the Norwegian Institute of Marine Research. All fish were cultured on the same diet (Nutra Olympic, Skretting) in 500L tanks with a water temperature of 10 °C. At approximately 100 g body weight the fish were bath challenged, where seawater flow was stopped for 1 h, oxygenation levels were controlled and 30 copepodids/fish were added to each tank. Once the copepodites reached the chalimus II stage the fish were euthanized humanely with a lethal dose of anaesthesia and manually counted for lice by a team of trained staff. Mucosal samples were collected by manual scraping and stored at − 80 °C. Control samples were taken at the start of the Gen trial only, including a single water sample from the water inlet and ten samples of mucus from mixed GenS and GenR fish prior to lice challenges.

The first challenge trial (Gen) used Benchmark Genetics fish which were offspring from two divergent lines which have undergone selection for resistance (GenR) and susceptibility (GenS) to salmon lice. Three families per line of 80 fish each were cultured at a standard water temperature 10 °C. The second challenge trial used the MOWI strain (50 families) with no artificial selection history for lice resistance, and were cultured at 10 °C and then gradually adjusted to the range of water temperatures (5 °C, 10 °C, 17 °C) for the lice challenge. Each water temperature had two tanks and the trial was run in duplicate (Supplementary Table 1). To ensure equal maturation of the copepodites to the chalimus II stage where they are still visible but not motile, across the different water temperatures, the termination date was adjusted using calculations based on the cumulative degree days (see Supplementary Table 1). From the Gen trial, four mucus samples were randomly selected from each of the six families in each of the two tanks (4 × 6 × 2). From the Temp trial, samples were ranked by lice count within tank and two high lice count and two low lice count samples were selected from each tank, in each water temperature from each of the trial replication (4 × 2 × 3 × 2). The low lice count samples had a mean lice count of 4 and the high lice count samples had a mean lice count of 41.

### Chemical analysis of volatile collections

Mucosal samples were thawed and ~ 100 µL of sample was transferred to a 20 mL headspace vial, flushed with nitrogen and capped with a teflon sealed screw cap. Volatile compounds were analysed using a Gerstel multipurpose sample automated dynamic head space system interfaced with an Agilent 7890B gas chromatograph (GC, Agilent, Palo Alto, CA, USA) and Agilent 5977B quadrupole mass selective detector. Incubation took place at 60 °C for 5 min under agitation followed by purging 200 mL gas volume over Tenax GR activated coal for trapping volatile compounds. An additional step (100 mL nitrogen at 30 °C for 16 min.) was used to remove trapped moisture from the adsorbent before the adsorbent tube was transferred to the thermal desorption unit of the GC injector port, where volatiles were desorbed at 10 °C/min up to 300 °C and transferred to the GC column kept at 30 °C. The compounds were separated on a DB-WAXetr column from Agilent (0.25 mm i.d., 0.5 µm film, 30 m) using a Helium (99.999%) carrier gas. The mass spectrometer was operated in electron impact (EI) mode at 70 eV ionization energy and measuring positive ion fragments. The MS scan rate was from m/z 33–500. Chemstation software (G1701CA version C.00.00, Agilent Technologies) was used to process the GC/MS output data. Identification of the compounds was confirmed by comparing the measured mass spectra of the GC peaks with pure standards according to the NIST015 mass spectrum library. GC integrated peak area were used as raw data for the VOC expression. In addition, blank controls and a sample tube were analysed to check for possible background contamination.

### Statistical analysis of volatiles and lice infectivity

The effect of water temperature on VOC expression levels was estimated using an ANOVA model of the following form:

1 $${y}_{ijkl}= {T}_{i}+{TEMP}_{j}+{G}_{k}+ {e}_{ijkl}$$

where ${y}_{ijkl}$ is the natural logarithm transformed VOC expression (1-penten-3-ol, 6-methyl-5-hepten-2-one or 1-octen-3-ol), *T* is the fixed effect of trial replicate (i = 2 levels), *TEMP* is the fixed effect of water temperature (j = 3 levels, 5 °C, 10 °C, 17 °C) and *G* is the fixed effect of lice group (k = 2 levels high lice count and low lice count) and e notes the random error term. Post-hoc pairwise t-tests where conducted controlling for multiple comparisons using the Benjamini–Hochberg procedure^48^.

The effect of divergent genetic line on lice count was estimated, but violated the statistical assumption of normally distributed residuals, a logarithmic base 10 transformation subsequently improved normality of residuals. The effect of genetic line was estimated using a nested-ANOVA model of the following form:

2 $${y}_{ijk}= {GEN}_{i}+{TANK}_{k}+ {e}_{ijk}$$

where ${y}_{ijk}$ is the logarithm base 10 transformed lice count (n = 171), GEN is the effect of divergent lice (i = two levels resistant, susceptible), TANK is the fixed effect of tanks (k = two levels) and e denotes the random error term. The effect of divergent genetic line on VOCs followed the same form as model 2 with VOCs expression levels natural logarithm transformed (benzene, 1-octen-3-ol, and 6-methyl-5-hepten-2-one).

The lice counts were further analysed using linear mixed models with average information restricted maximum likelihood across all lice challenged fish in populations Temp, Gen and combined (n = 5750) using DMU 6.5^49^. The model had the following form:

3 $${y}_{ijk}= {T}_{i}+{TTEMP}_{j}+ {a}_{k}+ {e}_{ijk}$$

where ${y}_{ijk}$ is logarithm 10 transformed lice count, ${T}_{i}$ is the fixed effect of trial where (i = 3 levels), TTEMP is the fixed effect of tank nested within temperature (j = 14 levels), ${a}_{k}$ is the random additive effect of the kth animal $\sim ND(0,{\varvec{A}}{\sigma }_{a}^{2})$ where **A** the pedigree-derived numerator relationship matrix and ${\sigma }_{a}^{2}$ is the additive genetic variance. The e is the random residual $\sim ND\left(0,\mathbf{I}{\sigma }_{e}^{2}\right)$, where I is the Identity matrix and ${\sigma }_{e}^{2}$ is the residual error variance. Narrow sense heritability was estimated as the ratio of additive genetic variance to the total phenotypic variance.

The broad sense heritability of VOCs was estimated using a similar linear mixed effect models as lice count except the variance structure between families $\sim ND(0,{\varvec{I}}{\sigma }_{f}^{2})$ was tested in place of the additive genetic variance. Narrow sense heritability was calculated as the ratio of additive genetic variance to total phenotypic variance whilst broad sense heritability was calculated as the ratio of between family variance and total variance.

Pearson’s correlation (R) was computed between the breeding value for lice count (model 3) and the corresponding VOC phenotypes. The genetic variation in lice count explained by the phenotypic variation in VOC phenotypes was calculated as the coefficient of determination.

## Supplementary Information

Supplementary Figure 1.

Supplementary Table 2.
