# Supplementary material for: Variation in volatile organic compounds in Atlantic salmon mucus is associated with resistance to salmon lice infection
Source: Sci Rep. 2022 Mar 22;12:4839. doi: 10.1038/s41598-022-08872-z (PMC8940922; doi:10.1038/s41598-022-08872-z)
Supplement: Supplementary file 1 — Supplementary Figure 1. [file 41598_2022_8872_MOESM1_ESM.docx]

3,5,5-trimethyl-2-hexene

High lice

Low lice

Control

Water

1-octen-3-ol
