# Supplementary material for: Variation in volatile organic compounds in Atlantic salmon mucus is associated with resistance to salmon lice infection
Source: Sci Rep. 2022 Mar 22;12:4839. doi: 10.1038/s41598-022-08872-z (PMC8940922; doi:10.1038/s41598-022-08872-z)
Supplement: Supplementary file 2 — Supplementary Table 2. [file 41598_2022_8872_MOESM2_ESM.docx]

Supplementary material

Supplementary table 1. Descriptive variables in size and temperature standardised design for in vivo lice challenges

| Trial | Tank | Water temperature (°C) | Cumulative degree days at Counting | Number of fish | Average weight (grams) ± SD | Average log_10_ (lice count) ± SD | Sampled for VOCs |
| --- | --- | --- | --- | --- | --- | --- | --- |
| Temp 1 |  |  |  |  |  |  |  |
|  | 1 | 5 | 155 | 465 | 99.1 ± 21.7 | 1.01 ± 0.24 | 4 |
|  | 2 | 5 | 160 | 438 | 96.5 ± 22.7 | 1.06 ± 0.26 | 4 |
|  | 3 | 10 | 140 | 456 | 96.7 ± 22.0 | 1.35 ± 0.24 | 4 |
|  | 4 | 10 | 150 | 423 | 103.1 ± 19.7 | 1.26 ± 0.22 | 4 |
|  | 5 | 17 | 102 | 443 | 103.7 ± 24.4 | 1.30 ± 0.20 | 4 |
|  | 6 | 17 | 119 | 439 | 101.0 ± 22.2 | 1.19 ± 0.19 | 4 |
| Temp 2 |  |  |  |  |  |  |  |
|  | 1 | 5 | 170 | 461 | 99.1 ± 22.3 | 0.98 ± 0.28 | 4 |
|  | 2 | 5 | 175 | 464 | 104.5 ± 21.1 | 1.10 ± 0.30 | 4 |
|  | 3 | 10 | 140 | 438 | 102.3 ± 19.2 | 0.99 ± 0.23 | 4 |
|  | 4 | 10 | 150 | 450 | 102.6 ± 23.4 | 0.98 ± 0.22 | 4 |
|  | 5 | 17 | 102 | 456 | 99.5 ± 19.5 | 1.12 ± 0.19 | 4 |
|  | 6 | 17 | 119 | 470 | 101.2 ± 23.0 | 1.15 ± 0.18 | 4 |
| Gen |  |  |  |  |  |  |  |
|  | 3 | 10 | 150 | 86 | 99.4 ± 19.4 | 1.38 ± 0.21 | 12 |
|  | 4 | 10 | 150 | 85 | 99.6 ± 18.0 | 1.43 ± 0.24 | 12 |
